# The Efficacy of Silver Diamine Fluoride as a Caries Preventive Agent on Permanent Teeth: A Scoping Review

**DOI:** 10.1055/s-0043-1776337

**Published:** 2024-02-08

**Authors:** Irmaleny Irmaleny, Kindys Zulva Phienna, Anna Muryani

**Affiliations:** 1 Department of Conservative Dentistry, Faculty of Dentistry, Universitas Padjadjaran, Bandung, Indonesia; 2 Undergraduate Program of Faculty of Dentistry, Universitas Padjadjaran, Bandung, Indonesia

**Keywords:** silver diamine fluoride, remineralize, dental caries, permanent teeth

## Abstract

Dental caries is a chronic condition that affects people of all ages and has a high prevalence in Indonesia. Applying silver diamine fluoride (SDF) as a topical fluoridation agent is one of the approaches to prevent bacterial interactions that lead to the development of carious lesions. This study aims to determine the efficacy of SDF as a caries preventive agent on permanent teeth. Article searches were conducted using the keywords “silver diamine fluoride” AND “permanent tooth OR permanent teeth” AND “caries OR dental caries” AND “remineralize” through digital databases including PubMed, Cochrane Library, Science Direct, and Google Scholar. Articles with randomized controlled trial or nonrandomized controlled trial designs, articles written in Indonesian or English, publications published within the last 5 years (2016–2021), and aligned with the PCC (population, concept, and context) framework were included. Articles that were not accessible in full text or in a paid format, those that were of the meta-analysis or systematic review study type, and those that did not evaluate the use of SDF as a caries preventive agent were excluded from consideration. This scoping review refers to the Preferred Reporting Items for Systematic Reviews and Meta-Analyses Extensions for Scoping Reviews (PRISMA-ScR) guidelines. A total of 8 articles were reviewed, encompassing various locations, designs, and samples, which demonstrated the use of SDF resulted in a high percentage of fluoride release with a high degree of remineralization efficacy. The deposition of crystals or minerals into fissures and crevices caused by demineralization indicated a reduction in lesion depth and influenced the microhardness of enamel. SDF can stimulate the rehardening of tooth structures rich in calcium and phosphate. The solution of SDF has the potential to serve as an alternative substance for preventing caries in permanent teeth because it enhances mineral precipitation and mineral density, promotes the remineralization of hydroxyapatite in enamel by increasing fluoride, and increases tooth structure resistance to acid attack.

## Introduction

Good oral and dental health is an essential part of an individual's overall well-being. 1 The condition of oral and dental health in Indonesia is still a matter of concern, thus requiring serious attention from health care professionals. The 2018 Basic Health Research (Riset Kesehatan Dasar or Riskesdas) reported that tooth decay, cavities, or pain are the most significant dental issues in Indonesia (45.3%), with a caries prevalence of 88.8%. As a result, it can be concluded that the prevalence of caries tends to be high (>70%) across all age groups. 2 According to the WHO Global Oral Health Status Report 2022, oral illnesses impact about 3.5 billion people globally, with three out of every four people affected living in middle-income countries. Caries of permanent teeth affects an estimated 2 billion individuals worldwide, with 514 million children suffering from caries of primary teeth. 3 Adult dental caries is widespread throughout the world, affecting nearly 100% of the population in the majority of countries. 4

Dental caries is a chronic disease that is preventable and caused by an imbalance between oral flora and dietary habits. This imbalance can lead to the development of biofilm on the hard tissues of the teeth. 1 5 6 Caries is a result of the interaction between cariogenic bacteria, microbial biofilm, and specific food components (especially low-molecular-weight carbohydrates). 7 Cariogenic foods contain fermented carbohydrates, and bacteria within the biofilm are metabolically active and capable of fermenting carbohydrates into acid within 1 to 3 minutes, causing a decrease in pH (pH < 5). Biofilm formation cannot be prevented, but disease progression can be controlled to prevent lesion formation. 5 8 9

A caries prevention or management program should consist of prophylactic therapy and interventions recommended based on the caries risk level, considering the patient's risk factors. 6 Prevention efforts for caries involve several factors that need to be modified, such as dietary habits, oral hygiene, fluoride use, fissure sealants, and the introduction of dental care at an early age. 10 11 Emphasis should be placed on restoring the balance of the oral environment as a protective mechanism for remineralization and repairing demineralization. 10 Caries prevention can be tailored to the individual characteristics of children and families based on caries risk assessment (CRA), caries diagnosis, and the choice of preventive agents such as fluoride varnish and silver diamine fluoride (SDF). 12

Fluoride varnish is one of the preventive agents that contain fluoride and can reduce the risk of caries in primary teeth by nearly one-third to almost half the number of permanent teeth. The application of fluoride varnish is recommended every 3 months (for patients, either children or adults, with high risk) to every 6 months (from the age of 3 to adolescence, adults with dry mouth, or active caries) to be more effective, although the required cost is relatively higher compared to SDF. 8 The use of SDF has been proven to arrest caries in animal models and is more effective than sodium fluoride varnish in human trials. 13 This is because sodium fluoride varnish works well in preventing new lesions and addressing noncavitated lesions, while SDF is used to halt the progression of caries at the stage of cavity formation. 14

SDF is a topical fluoride solution that has been approved by the Food and Drug Administration (FDA) of the United States 12 as an antihypersensitivity agent and is used to arrest caries lesions 12 15 by inhibiting caries development and suppressing biofilm formation. 6 13 15 16 17 18 19 20 21 Research on the application of SDF in school environments indicates that SDF is safe for use 12 and has a simple application procedure, making it applicable directly to the tooth surface. Furthermore, SDF is relatively cost-effective. 15 22 The application of SDF does not lead to abscesses in treated carious teeth, does not cause pain, and has no side effects on patients without silver allergies. 13 15 19 However, SDF may leave black stains on treated caries lesions, due to the formation of silver phosphate, 18 23 while sodium fluoride varnish can induce nausea in individuals with gastric acid issues. 24 Due to the limited penetrating capacity of the agent that operates in the enamel external area, this minimally invasive technique does not fix the aesthetic problem in advanced lesions and may result in an untreated discolored area. 25 Therefore, the use of SDF should be considered in cases related to aesthetics. 26 Despite the higher efficacy of SDF in clinical trials when compared to other nonsurgical treatments, the ensuing black stain is an unsettling side effect that impairs patients' or parents' acceptance of this treatment. 27

A high prevalence of caries necessitates intervention before the condition worsens. However, on the other hand, patients also need to be aware of the impact of caries formation in order to effectively manage and prevent it. Nowadays, several clinical trials have been conducted in Asia and United States regarding the use of SDF as a substance that can arrest the growth of caries lesions in children's teeth. 28 29 30 31 32 33 34 35 This scoping review aims to determine the efficacy of SDF as a caries preventive agent on permanent teeth.

## Methods

The research methodology used in this article is a scoping review. The research questions are determined in line with the writing objectives, guided by the PCC (Population, Concept, and Context) 36 framework as follows: (1) Population—males and females aged ≥6 years with permanent teeth and without caries; (2) Concept—the topical use of SDF as a caries preventive agent; (3) Context—Original research articles of the randomized controlled trial and nonrandomized controlled trial study types.

Article screening was conducted systematically following the Preferred Reporting Items for Systematic reviews without Meta-Analyses extension for Scoping Reviews (PRISMA-ScR) guidelines. 37 The digital databases used were PubMed, Cochrane Library, Science Direct, and Google Scholar. Additional article searches were performed by examining the reference lists of the obtained articles (manual handsearching) and were included if relevant to the research topic. The search strategy in this research used “Boolean Operators” (“AND,” “OR,” and “NOT”) to combine or exclude keywords, yielding more focused and productive results. The research question was carried out using the keywords “(silver diamine fluoride) AND (permanent tooth OR permanent teeth) AND (caries OR dental caries) AND (remineralize).” SDF is used as a keyword since there has been little discussion on prevention, and it is typically used to prevent caries in permanent teeth. Meanwhile, it is commonly used as a remineralization agent to prevent caries in children. The inclusion criteria used in the study were articles of the randomized controlled trial and nonrandomized controlled trial study types in both Indonesian and English languages, published within the last 5 years (2016–2021), involving male and female subjects aged ≥6 years with permanent teeth and without caries. The exclusion criteria included articles that were not accessible in full text and in paid format, articles of the meta-analysis or systematic review study types, and those that did not assess the use of SDF as a caries preventive agent. The obtained articles were further screened by removing duplicates and reviewing the topic, title, and abstract. Subsequently, the filtered articles were reviewed by reading the full text content, aligned with the inclusion and exclusion criteria, to be included in the data extraction table.

## Results

**Fig. 1:**
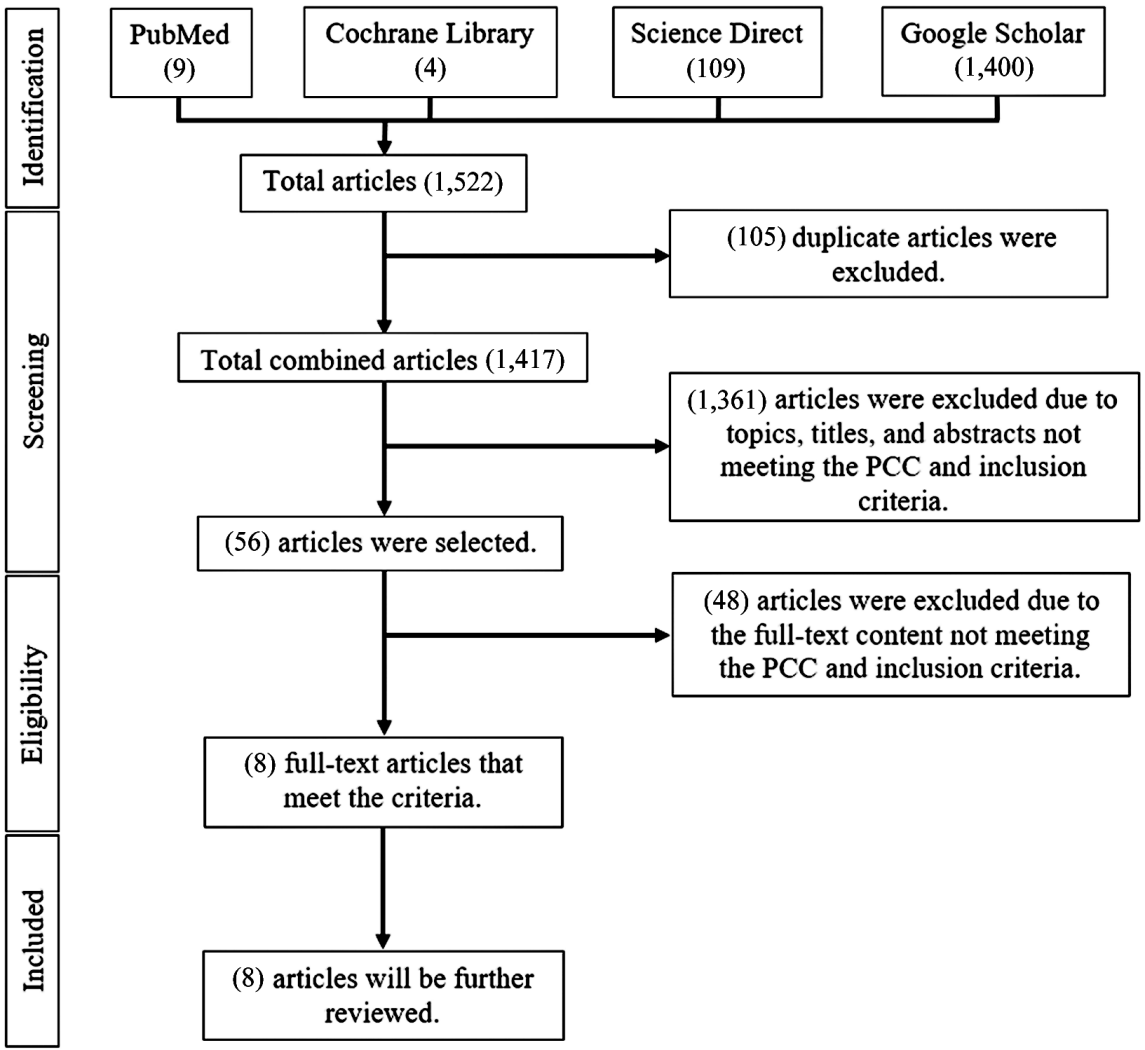
Research procedure stages.

Literature search using keywords across the four databases yielded 1,522 articles, with the following breakdown: 9 articles from PubMed, 4 articles from Cochrane Library, 109 articles from ScienceDirect, and 1,400 articles from Google Scholar. The first screening phase involved removing 105 duplicate articles, resulting in 1,417 articles. The second screening phase involved reviewing the relevance of topics, titles, and abstracts, aligning them with the PCC criteria and inclusion criteria. This led to the selection of 56 articles, while 1,361 articles were excluded. The third phase, or eligibility criteria screening, involved reading the full-text content of articles. This resulted in eight articles for further assessment, while 48 articles were excluded as they did not match the PCC and inclusion criteria. The article screening process is depicted in Fig. 1 of the research procedure stages within the execution diagram using the PRISMA-ScR framework. The eight reviewed articles were then entered into a data extraction table ( Table 1 ). The data extraction table summarizes the data, including researcher names, study years, titles, locations, designs, samples, and research outcomes.

**Table 1:** Data extraction results

Sl. no.	Study	Title	Location	Design	Sample	Research outcome
**1**	Yu et al 47	Remineralisation of enamel with silver diamine fluoride and sodium fluoride	China	Randomized controlled trial	Forty-eight enamel specimens (4 × 2 × 2 mm ^3^ ) obtained from 12 extracted molars, divided into 4 groups: 1. Group 1: silver diamine fluoride (SDF) 38% + sodium fluoride (NaF) varnish 5% ( *n* = 12) 2. Group 2: SDF 38% ( *n* = 12) 3. Group 3: NaF varnish 5% ( *n* = 12) 4. Group 4: deionized water (negative control; *n* = 12)	The enamel surface treated with both SDF and NaF appeared denser and more intact compared to other groups There was no significant difference in fluoride content between the application of SDF and NaF ( *p* = 0.073) Groups treated with SDF (groups 1 and 2) exhibited a higher fluoride content compared to groups without SDF (groups 3 and 4; *p* < 0.001) Groups 1 and 2 showed shallower lesion depths compared to group 3 ( *p* = 0.002) and group 4 ( *p* < 0.001) Hydroxyapatite diffraction peaks were detected in all four groups, but strong diffraction reflections were observed in the SDF-treated groups (groups 1 and 2). These reflections were confirmed as AgCl (silver chloride) through X-ray diffraction Nanoparticles with a diameter of around 5 nm were detected on hydroxyapatite crystals in the SDF-treated group. These nanoparticles were confirmed to be silver through energy dispersive spectroscopy (EDS) Clear peaks of Ag3d in X-ray photoelectron spectroscopy (XPS) were observed in the SDF-treated group, indicating that the silver on hydroxyapatite was in the form of metallic silver
**2**	Idorasi et al 42	Morphological aspects in remineralizing potential of silver diamine fluoride	Romania	Nonrandomized controlled trial	Four extracted teeth, sectioned mesiodistally into two parts ( *n* = 8)	Significant difference in SDF penetration percentage for proximal areas compared to occlusal areas ( *t* = 3.161, *p* = 0.003 to <0.05)
**3**	Farhadian et al 50	The effect of silver diamine fluoride versus sodium fluoride varnish on the microhardness of demineralized enamel: an *in vitro* study	Iran	Randomized controlled trial	Sixty extracted premolars, divided into 4 groups: 1. Group 1: no intervention (control; *n* = 15) 2. Group 2: demineralization solution ( *n* = 15) 3. Group 3: NaF varnish 5% ( *n* = 15) 4. Group 4: SDF 38% ( *n* = 15)	Significant increase in microhardness of enamel surface in the NaF and SDF groups compared to demineralized enamel group ( *p* = 0.001 and <0.001, respectively) A statistically significant difference was observed in the average microhardness value of the enamel surface between the SDF-treated group and the NaF-treated group, with the SDF group having a higher microhardness value ( *p* = 0.004) Teeth treated with SDF exhibited a change in enamel color (from brown to grayish) a few days after application. This color change was attributed to the presence of silver components within the SDF solution
**4**	Saad et al 54	The effect of silver diamine fluoride on surface characterization of demineralized dentin	Egypt	Randomized controlled trial	Forty extracted molars, sectioned mesiodistally into 2 parts ( *n* = 80), divided into 2 main groups with 2 subgroups each: 1. Main group 1: demineralized dentin surface ( *n* = 40): a. Subgroup 1a: SDF ( *n* = 20) b. Subgroup 1b: no SDF ( *n* = 20) 2. Main group 2: healthy dentin surface ( *n* = 40) a. Subgroup 2a: SDF ( *n* = 20) b. Subgroup 2b: no SDF ( *n* = 20)	In specimens of healthy dentin, a longitudinal section of the dentin showed a smear layer covering it, and there was a deposition of SDF on the walls of dentin tubules In specimens of demineralized dentin, a longitudinal section of the dentin revealed an open dentin tubule structure with a deposition of SDF inside the dentin tubules
**5**	Srisomboon et al 45	Effects of different application times of silver diamine fluoride on mineral precipitation in demineralized dentin	Thailand	Randomized controlled trial	Thirteen upper jaw molars, sectioned horizontally and perpendicular to dentinal tubules at ∼2mm below occlusal surface to obtain dentin sections ( *n* = 52), divided into 4 groups: 1. Group 1: Deionized water (control; *n* = 13) 2. Group 2: 30-s SDF ( *n* = 13) 3. Group 3: 60-s SDF ( *n* = 13) 4. Group 4: 180-s SDF ( *n* = 13)	A statistically significant difference was observed in the Abs1024/Abs1636 ratio from 0 h to 2 wk in the control group (–0.035), which was lower compared to the 30-s group (0.077), 60-s group (0.068), and 180-s group (0.106). The values in the 30-, 60-s, and 180-s groups were similar ( *p* > 0.05) Dentin peritubular and dentin tubules in the specimens from the 30-, 60-, and 180-s groups were filled with crystal precipitations containing calcium (Ca), phosphorus (P), and fluorine (F). Additionally, crystals composed of silver (Ag) and chlorine (Cl) were also observed Demineralized specimens treated with SDF exhibited scattered radio dense clusters (precipitated minerals) in the demineralization layer The degree of mineral precipitation (mineral density, vol%) in the 30-s (65.6 ± 7.5 vol%), 60-s (65.8 ± 2.0 vol%), and 180-s (65.2 ± 8.0%) groups was comparable ( *p* > 0.05).
**6**	El Soud et al 49	Comparative Evaluation of the effects of silver diamine fluoride (commercial and lab prepared) versus nano silver fluoride on demineralized human enamel surfaces ( *in vitro* study)	Egypt	Randomized controlled trial	Twenty extracted premolars, sectioned buccolingually into 2 parts ( *n* = 40), divided into 5 groups: 1. Group 1: no treatment (negative control; *n* = 8) 2. Group 2: demineralization solution ( *n* = 8) 3. Group 3: commercial SDF ( *n* = 8) 4. Group 4: Lab-prepared SDF ( *n* = 8) 5. Group 5: nano silver fluoride ( *n* = 8)	There was a reduction in the number of inter crystalline gaps in group 3, accompanied by the formation of small crystals distributed on the enamel surface. A distinct “keyhole” appearance was observed in this group, which was characteristic and different from group 2 (the demineralization group). This indicates that the application of SDF led to changes in the enamel surface morphology and the distribution of crystals Scanning electron microscopy (SEM) examination of specimens in group 4 revealed the penetration of SDF particles into fissures and cracks that had formed due to demineralization. This suggests that SDF particles were able to infiltrate these microstructures Statistical analysis indicated a significant difference with a *p* -value of 0.05 for the ratios of phosphorus (P), calcium (Ca), and the Ca/P ratio. The highest content of phosphorus was observed in groups 5 and 4, indicating the potential effect of SDF and other substances on the mineral composition of the specimens
**7**	Tripathi et al 43	Evaluation of remineralizing capacity of P11-4, CPP-ACP, silver diamine fluoride, and NovaMin: an *in vitro* study	India	Randomized controlled trial	Sixty extracted premolars, divided into 4 groups: 1. Group 1: self-assembling peptide (P11-4; *n* = 15) 2. Group 2: SDF 38% ( *n* = 15) 3. Group 3: Casein phosphopeptide-stabilized amorphous calcium phosphate (CPP-ACP; *n* = 15) 4. Group 4: NovaMin ( *n* = 15)	The maximum value in the analysis of variance (ANOVA) test was obtained on the 21st day of the experiment. There was a significant difference in remineralization efficacy among the groups. The highest efficacy of remineralization was observed in group 1, followed by groups 3, 2, and 4. This suggests that group 1 showed the most effective remineralization, and there were varying levels of effectiveness in the other groups There was a statistically significant difference in the average remineralization values between groups 1 and 2 ( *p* < 0.05). This indicates that group 1 exhibited a higher level of remineralization compared to group 2, and the difference between their remineralization effects was considered significant from a statistical perspective
**8**	Thakur et al 48	Comparison of the fluoride release of silver diamine fluoride, fluoride varnish, acidulated phosphate fluoride gel on extracted teeth over various time intervals in artificial saliva	India	Randomized controlled trial	Ninety-six extracted premolar teeth were divided into four groups as follows: 1. Group 1: SDF 38% ( *n* = 24) 2. Group 2: acidulated phosphate fluoride (APF) gel 1.23% ( *n* = 24) 3. Group 3: bifluoride 10 varnish ( *n* = 24) 4. Group 4: no treatment (negative control; *n* = 24)	There was a statistically significant difference in the average fluoride release at the time intervals of 24 h, 7 d, and 14 d among all four groups ( *p* < 0.001). This suggests that the amount of fluoride released varied significantly across the different groups at different time points There was also a statistically significant difference in the average fluoride release from the SDF group at the time intervals of 24 h, 7 d, and 14 d. This implies that the SDF group exhibited a distinct pattern of fluoride release compared to other groups, and this difference was statistically significant

**Fig. 2:**
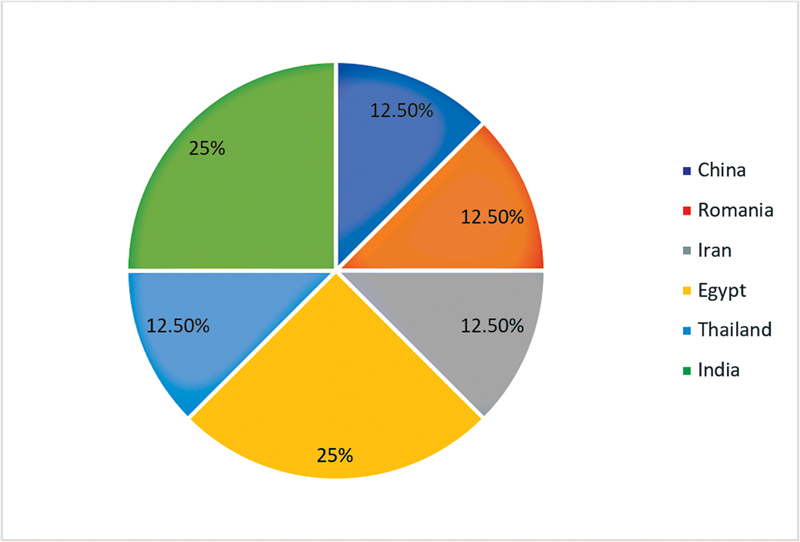
The research was conducted in six different countries.

**Fig. 3:**
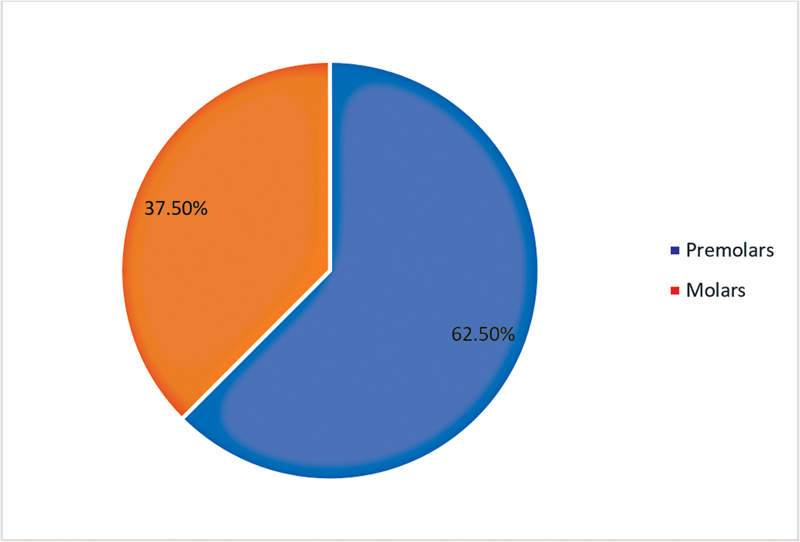
The variety of teeth that are being used.

**Fig. 4:**
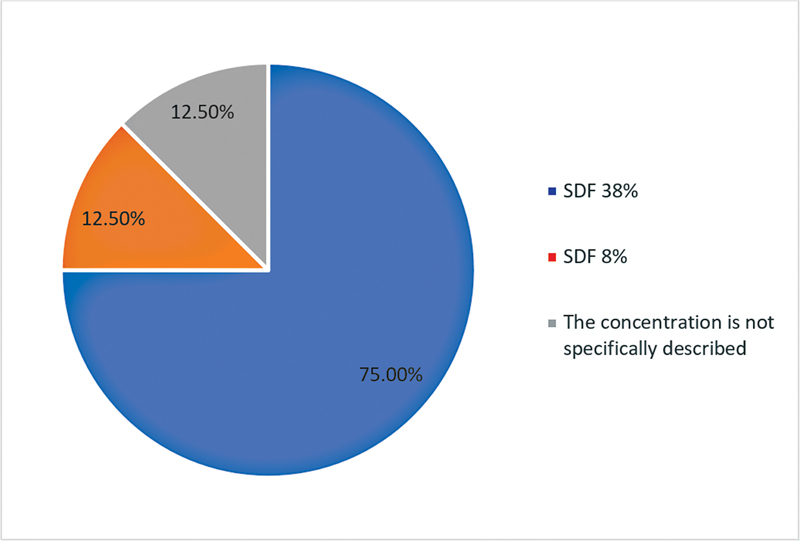
The concentration of the solution utilized. SDF, silver diamine fluoride.

**Fig. 5:**
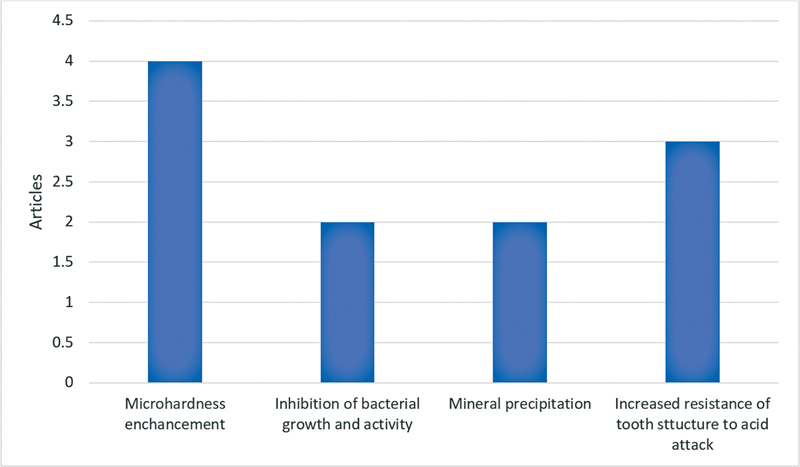
The article's findings after using silver diamine fluoride (SDF).

Based on Table 1 , the key findings from the eight research articles related to the efficacy of SDF as a caries preventive agent on permanent teeth. The studies covered six countries ( Fig. 2 ): China (1 article, 12.5%), Romania (1 article, 12.5%), Iran (1 article, 12.5%), Egypt (2 articles, 25%), Thailand (1 article, 12.5%), and India (2 articles, 25%). Age and gender information about the study participants was not mentioned in any of the eight articles. Tooth types used as samples varied ( Fig. 3 ): premolars were used in five articles (62.5%), and molars were used in three articles (37.5%). Different concentrations of SDF were used ( Fig. 4 ): 38% SDF was used in six articles (75%), 8% SDF was used in one article (12.5%), and one article (12.5%) did not specify the concentration used. Remineralization improvement after SDF application was reported in all eight articles. Microhardness enhancement was reported in four articles (50%). Inhibition of bacterial growth and activity was reported in two articles (25%). Mineral precipitation was reported in two articles (25%). Increased resistance of tooth structure to acid attack was reported in three articles (37.5%; Fig. 5 ).

## Discussion

Fluoride use has reduced caries prevalence in the last 50 years; however, considering dental caries remains a substantial concern for some children and adults, management of this health condition has changed. CRA, reducing or eliminating certain organisms (mainly *Streptococcus mutans* and *Lactobacillus* ), preventing demineralization, and improving remineralization are all part of the prevention strategy. 38 Demineralization is the process of removing mineral ions from the hard tissue of teeth, such as enamel, due to an increase in hydroxyapatite concentration on the tooth surface caused by acid attack. 39 40 This often occurs due to bacterial biofilm–produced acids. The damage to enamel caused by acid production from bacteria can be restored through remineralization, which involves the use of fluoride to enhance the conversion of hydroxyapatite crystals into more stable fluorohydroxyapatite or fluorapatite crystals that are less soluble in acids. 41 SDF solution contains antibacterial and remineralization agents that can improve tooth resistance to acid attacks by forming Ag-protein conjugates on damaged enamel surfaces. 42 SDF has the highest concentration (44,800 ppm) 43 44 and can promote the absorption of calcium and phosphate, resulting in the formation of apatite minerals on the enamel surface, which inhibits demineralization and increases microhardness values. 45 46 The concentration of fluoride plays a role in its absorption and retention. 43 Higher concentrations of fluoride are bactericidal, while lower concentrations promote remineralization. However, both high and low concentrations lead to the formation of fluorapatite crystals, inducing the precipitation of fluorapatite mineral phases within the tooth structure. 41

The samples in the eight studied articles underwent pH cycling, and it was reported that there was an increase in remineralization efficacy after applying SDF. The study by Yu et al on 12 tooth specimens treated with 38% SDF and 12 tooth specimens treated with 38% SDF + 5% NaF varnish showed a high percentage of fluoride release compared to the group without SDF. 47 This is consistent with the findings of Thakur et al, which showed significant differences in fluoride release at 24 hours, 7 days, and 14 days after SDF application. 48 The addition of SDF + NaF resulted in similar levels of remineralization based on lesion depth and the mineral density value (MDV) of enamel. This similarity occurs because the availability of calcium ions on the tooth surface is limited. When the amount of fluoride exceeds the amount of calcium, excess fluoride does not contribute to remineralization and can even lead to fluorosis. 39 47 The SDF-treated group exhibited hydroxyapatite enamel remineralization due to a significant increase in fluoride on the demineralized enamel surface. 49 This aligns with the results of the study conducted by Tripathi et al, which noted an improvement in the remineralization efficacy of SDF on days 7, 14, and 21. 43

The study by Farhadian et al compared the results of applying SDF and sodium fluoride varnish on 60 premolar teeth. In this study, it was reported that teeth treated with SDF exhibited a change in enamel color to brown-gray a few days after application, which was caused by the silver component of SDF. 50 This is consistent with the findings of Tripathi et al, who stated that color changes after SDF application are influenced by the deposition of silver phosphate followed by an increase in phosphate ions. 43 The deposition of silver phosphate on demineralized enamel surfaces as well as caries lesions is the cause of the appearance of black stains after SDF application. Precipitation of silver sulfite can occur through a reaction between the silver compounds present in SDF and sulfur compounds found in saliva, food residues, or specific microorganisms in the oral cavity, which can contribute to staining. Black stains on dentin caries become visible within 2 minutes after application, and significant visual changes occur within 5 minutes. Staining on enamel and surrounding tissues becomes noticeable within 4 to 6 hours after SDF application. 27 Even though SDF application can cause black stains, SDF is still regarded as an alternative therapy, recommended for cases such as primary teeth, permanent teeth that do not require high aesthetic demand, severe anxiety (of dental therapy), and root caries in geriatric patients. 51 52 53

The study by Saad et al reported that the application of SDF on 20 specimens of healthy dentin showed a longitudinal section of dentin covered by a smear layer with SDF deposition on the dentin tubule walls. On the other hand, the application of SDF on 20 specimens of demineralized dentin showed a longitudinal section of dentin with SDF deposition inside the exposed dentin tubules. 54 This is consistent with the findings of Farhadian et al, who explained that SDF solution can be used for dentin treatment by forming a layer of Ag-protein conjugate on the dentin surface, which then precipitates inside the dentin tubules. 50 Demineralized dentin has a larger number of open collagen fibers, which affects the amount of silver ion absorption because the soluble silver from SDF has strong bonding to collagen, resulting in the removal of the smear layer and causing deeper deposition of silver particles within the dentin tubules. 54 55

The study conducted by Srisomboon et al on 52 demineralized dentin specimens detected crystal deposits in the peritubular dentin and dentin tubules containing Ca, P, and F. Additionally, crystals consisting of Ag and Cl were also observed in their research. 45 This aligns with the findings of Yu et al, who detected the peak diffraction of hydroxyapatite in their study. Strong diffraction reflections were detected in the group with SDF treatment, confirmed as silver chloride (AgCl) through X-ray diffraction (XRD). Nanoparticles with a diameter of ± 5 nm detected on the hydroxyapatite crystals in the SDF-treated group were confirmed as silver using energy dispersive spectroscopy (EDS). X-ray photoelectron spectroscopy (XPS) results showed that the silver on hydroxyapatite was metallic silver. 47 The interaction between hydroxyapatite and SDF led to the formation of limited quantities of nanoscale metallic silver particles that adhered to the hydroxyapatite crystals. 56 Silver phosphate and silver oxide are formed when silver nitrate reacts with hydroxyapatite, and then silver oxide reacts with an alkaline chloride solution to precipitate AgCl. 57

The study by El Soud et al demonstrated that in the group treated with laboratory-made SDF, particles of SDF penetrated into fissures and cracks resulting from demineralization. The group treated with commercial SDF showed a reduction in the number of intercrystalline gaps, forming small crystals distributed on the enamel surface, leading to the typical appearance of a keyhole. 49 This is in line with the research conducted by Srisomboon et al, where specimens treated with SDF showed peritubular dentin and dentin tubules filled with precipitated crystals with scattered radio-dense clusters. 45 Demineralized dentin retains some hydroxyapatite, which can act as apatite nucleation sites, allowing remineralization and restoration of mechanical properties. 57 The fluoride ions in SDF can facilitate silver phosphate to restore mineral content. 43 A dense granular structure is observed on the surface of demineralized dentin, indicating mineral formation outside collagen fibers. 57

The study conducted by Srisomboon et al on 52 demineralized dentin specimens treated with the application of 38% SDF for 30, 60, and 180 seconds showed comparable degrees of mineral precipitation (mineral density). 45 This is consistent with the research by Idorasi et al, which explains that the components within SDF can enhance mineral density by increasing hydroxyapatite and fluorapatite. 42 The sharper diffraction reflections of hydroxyapatite found by Yu et al in the SDF + NaF, SDF, and NaF groups indicate better crystallization of hydroxyapatite compared to the deionized water group. 47 SDF can increase mineral precipitation in lesions up to 150 μm and enhance mineral density, thus aiding in reducing lesion depth from ± 250 to ± 100 μm. 45 The fluoride ions present in SDF can facilitate the precipitation of calcium fluoride; when the size of calcium fluoride crystals is larger, the resistance to acid increases, leading to minimized demineralization effects. 58

Microhardness of the enamel surface is one of the methods used to test the enamel's resistance after the application of fluoride agents. 59 In the study by Farhadian et al, involving 15 premolar tooth specimens each treated with 38% SDF and NaF, significant differences in microhardness of the enamel surface were observed compared to the demineralized enamel group. Teeth treated with SDF exhibited higher potential for remineralization compared to NaF and demonstrated microhardness of the enamel surface comparable to healthy enamel. 50 This is in line with the findings of the study by Yu et al, which stated that the enamel surface treated with SDF and NaF remained relatively dense and intact compared to other groups. 47 Remineralization is a crucial process that significantly influences enamel hardness and strength. 39 Topical fluoride application leads to the formation of a layer of calcium fluoride, which subsequently diffuses to the enamel surface to form fluorapatite, thus aiding in enhancing remineralization and enamel hardness. 46 SDF can penetrate more easily on proximal surfaces compared to occlusal surfaces due to the smoother topographical nature of the proximal surface and the absence of pits and fissures. 42

SDF is a topical fluoride solution that has the ability to inhibit the metabolism of *S. mutans* , which is the main cause of dental caries, 40 60 and promote the remineralization of hydroxyapatite in enamel. 47 49 This is in line with the alkaline nature of SDF, which facilitates the precipitation of fluorapatite to maintain collagen as a foundation for mineral precipitation. 49 The application of topical fluoride on enamel induces the formation of unstable fluoride (calcium fluoride) as a reservoir on the enamel surface. Soluble calcium fluoride deposits at an acidic pH (<5) will stably bind to form fluor-hydroxyapatite, 47 61 thereby stimulating remineralization in both enamel and dentin 48 62 63 and helping strengthen enamel's resistance to demineralization. This, in turn, reduces and prevents the future occurrence of caries. 23 63

As a dental practitioner, it is important to know the methods to improve tooth hard tissue biomechanical properties in the conservation of dentition. This scoping review study indicates varied outcomes from each reviewed article. The results of the eight articles complement each other regarding the efficacy of SDF as a caries preventive agent on permanent teeth. Limitations in information concerning the differences in SDF concentration formulations, age of the samples, and gender of the participants used can be a challenge when comparing the effectiveness of SDF among the reviewed articles. The review did not encompass all continents worldwide; it only included four countries in Asia, one country in Africa, and one country in Europe. Further recommendations could involve conducting additional clinical trials on topical SDF formulations with various dosages across different age groups. This would strengthen the evidence-based utilization of SDF in preventing caries in permanent teeth and could present an opportunity for further research on a broader country or continent scale.

The results of this article are expected to serve as evidence-based support for clinical considerations of SDF application as a caries prevention agent for permanent teeth. Furthermore, it is hoped that these findings will form the basis for further research and clinical trials.

## Conclusion

The efficacy of SDF as a caries preventive agent on permanent teeth can stimulate the rehardening of tooth structure rich in calcium and phosphate by enhancing mineral precipitation and density, promoting the remineralization of enamel hydroxyapatite by increasing fluoride, and making the tooth structure resistant to potential acid attacks. This suggests that SDF could be an alternative material for preventing caries in permanent teeth.
